# Stimulus-Responsive Nanomedicines for Disease Diagnosis and Treatment

**DOI:** 10.3390/ijms21176380

**Published:** 2020-09-02

**Authors:** Gengqi Liu, Jonathan F. Lovell, Lei Zhang, Yumiao Zhang

**Affiliations:** 1 School of Chemical Engineering and Technology, Tianjin University, Tianjin 300350, China; Gengqi_Liu@tju.edu.cn; 2 Key Laboratory of Systems Bioengineering (Ministry of Education), Tianjin University, Tianjin 300072, China; 3 Department of Biomedical Engineering, The State University of New York at Buffalo, Buffalo, NY 14260, USA; jflovell@buffalo.edu

**Keywords:** stimulus-responsive, chemical structures, drug conjugates, drug delivery, molecular imaging

## Abstract

Stimulus-responsive drug delivery systems generally aim to release the active pharmaceutical ingredient (API) in response to specific conditions and have recently been explored for disease treatments. These approaches can also be extended to molecular imaging to report on disease diagnosis and management. The stimuli used for activation are based on differences between the environment of the diseased or targeted sites, and normal tissues. Endogenous stimuli include pH, redox reactions, enzymatic activity, temperature and others. Exogenous site-specific stimuli include the use of magnetic fields, light, ultrasound and others. These endogenous or exogenous stimuli lead to structural changes or cleavage of the cargo carrier, leading to release of the API. A wide variety of stimulus-responsive systems have been developed—responsive to both a single stimulus or multiple stimuli—and represent a theranostic tool for disease treatment. In this review, stimuli commonly used in the development of theranostic nanoplatforms are enumerated. An emphasis on chemical structure and property relationships is provided, aiming to focus on insights for the design of stimulus-responsive delivery systems. Several examples of theranostic applications of these stimulus-responsive nanomedicines are discussed.

## 1. Introduction

**Figure 1:**
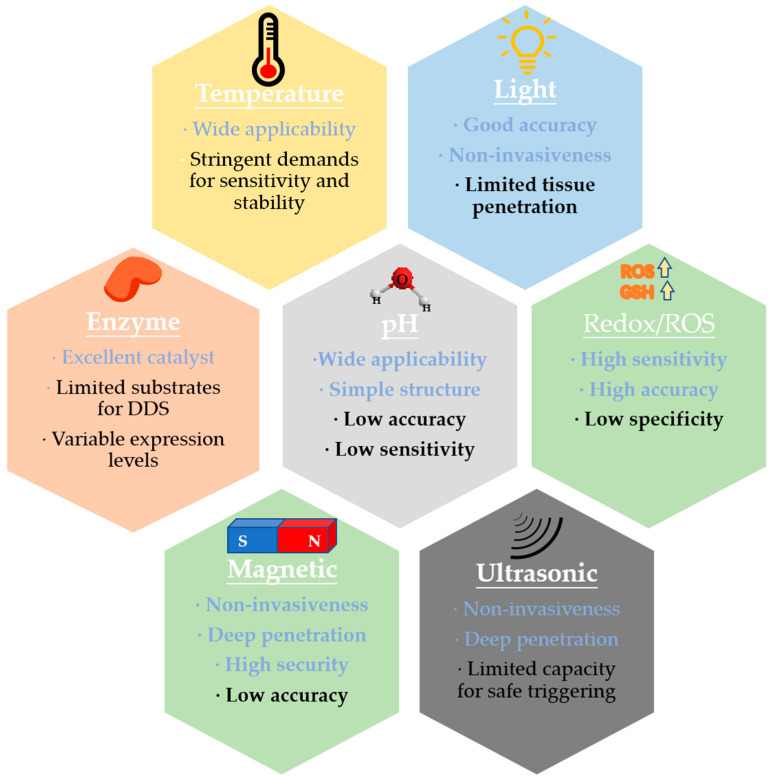
A list of various stimuli used for stimulus-responsive systems, along with advantages and disadvantages.

One of the challenges of traditional drug delivery systems is the lack of specific targeting capability, which leads to dose-limiting side effects [1,2]. Some drugs can be rapidly metabolized, and low bioavailability necessitates large injection doses but unsatisfactory therapeutic effects. For example, it is generally necessary to increase the dosage of anticancer drugs for the desired therapeutic effect, to an extent that also causes undesirable side effects [3,4]. In addition, orally administered medicines need to evade the risk of degradation in the acidic/enzymatic environment in the gastrointestinal tract [5,6]. All of these problems can potentially be addressed with stimulus-responsive nanotechnology. Utilizing specific microenvironmental conditions at diseased sites or alternatively by using external stimuli, nanotherapeutics can release active therapeutic ingredients specifically at desired spots in a controlled and targeted manner. As early as 1978, Tanaka described the phase transition of polyacrylamide polymers [7], and in the same year Blumenthal reported thermosensitive liposomes [8]. Findings such as these laid the groundwork for stimulus-responsive drug delivery systems, which have been developed for more than 40 years. Stimulus-responsive nanomaterials have been designed for a wide variety of different delivery or release purposes. The advantages and disadvantages of various stimulus-responsive systems are summarized in Figure 1.

Differences between the microenvironment of diseased tissues (e.g., arthritic tissues, tumor cells, and other diseases) and normal tissues can be used as endogenous stimuli to trigger drug release. Endogenous stimuli include lower pH values in tumor cells, high concentrations of glutathione (GSH) and reactive oxygen species (ROS) in inflammatory tissues and cancer tissues, and specific enzymatic activity at the target sites [9,10]. In addition to these endogenous stimuli above, exogenous stimuli such as magnet, light, and ultrasound can realize real-time tracking of drug distribution, disease diagnosis and disease management. Stimulus-responsive systems responsive to one single stimulus or multiple stimuli have been used to treat or diagnose a variety of diseases, such as cancers (colon cancer [11,12], breast cancer [13,14], lymphoma [15,16]), inflammatory diseases (inflammatory bowel disease [17,18], neurodegenerative diseases [19]), diabetes [20,21] and others. Some nanomedicines that could be considered stimulus-responsive have successfully gone into clinical trials. ThermoDOX (thermosensitive doxorubicin liposomes) used heat to release the cargo from liposome [22]; other successful examples include Opaxio [23] (involving on enzyme-degradable polymer), Nanotherm [24] (based on magnetic iron oxide nanoparticles), and Auroshell [25] (based on thermosensitive gold nanoshells). Stimulus-responsive systems also have potential for bioimaging. They can enhance the contrast in specific diseased area in response to internal or external stimuli for real-time monitoring.

In this review, we summarize stimulus-responsive nanomedicines, starting with a brief discussion of each stimulus, then various chemical structures that are sensitive to different stimuli are highlighted. Application examples of stimulus-responsive nanomedicines are discussed.

## 2. Stimulus-Responsive Systems

### 2.1. pH Stimulus

pH has been used for the design of stimulus-responsive systems because the value of pH varies among different subcellular locations and can differ between normal and diseased tissues. For example, due to the high glycolysis rate of cancer cells under partial anaerobic conditions, the pH value of cancerous tissues is lower than normal [26,27]. Tumors exhibit an acidic pH range of 5.7 to 7.8, while corresponding not-carcinogenic tissues usually have a pH close to 7.4 [28,29,30]. Similarly, it has also been shown that the pH value can decrease to 6.5 sixty hours after the onset of an inflammatory process [31]. In addition, different pH values can also be found in organelles, such as in endosomes and lysosomes with a pH range of from 4.5 to 5.5. Thus, many stimulus-responsive therapy systems have been designed that can remain stable in normal tissues, but release cargo in diseased tissues with structural changes responsive to pH changes [32]. The development of pH-responsive systems is mainly based on polymers with weak acids (such as carboxylic acid) or bases (such as primary amine and tertiary amine) groups, which can realize sharp change of ionization states in the desired pH range. The increase of ionization degree can significantly change the conformation of the chain and the affinity for the solvent, leading to the cleavage or change of the overall conformation. Additionally, the pH-responsiveness can be easily adjusted by changing the properties of monomers used in polymers [33].

The first type of pH-responsive functional groups are basic groups such as amines, pyridines and imidazoles (Table 1). For example, Liu et al. synthesized nanoscale coordination polymers (NCPs) based on hafnium (Hf) ions and pH-responsive benzoic acid imine linker. Excellent compatibility and stability were achieved after further modification by polyethylene glycol (PEG). Hf ions can also enhance the effect of radiotherapy. In the acidic environment of tumors, the chemotherapeutic drug chlorine (triphenylphosphine) gold (I) (TPPGC) could be released due to the degradation of benzoic acid imine linker, which gave rise to the damage of mitochondria of cancer cells and their apopsis [34]. Other types of functional group include hydrazone bond [35,36], glycerol ester groups [37], acetal [38,39], cis-acotinyl [40], orthoester [41,42], and silyl ether [43], which are unstable under acidic conditions. These chemical bonds could be hydrolyzed rapidly under weak acidic conditions, thereby causing the changes of the chemical structures (Table 1). In addition, acetal is one of the most widely used functional groups. The pH stimulus-responsive system based on acetal has the advantages of fast pyrolysis and minimal by-products. Recently, it has been reported that poly (ethylene glycol) and paclitaxel could be linked using acetal. Additionally, this conjugate can be self-assembled to form pH stimulus-responsive micellar nanoparticles. With a high drug loading of 60.3%, the nanoparticles could maintain high stability in normal tissues, but once delivered in tumor, the drug can be effectively released because of the fast pyrolysis of the acetal group [44].

**Figure 2:**
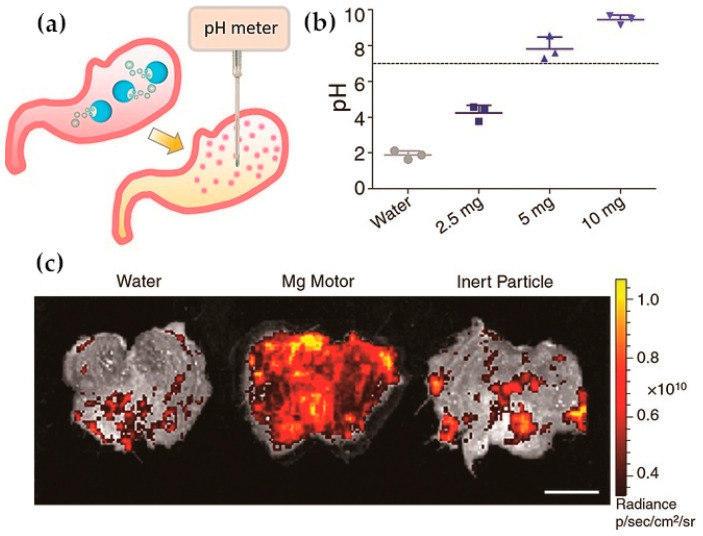
pH-stimulated cargo release. (**a**) Schematic illustration of the process of gastric acid neutralization by magnesium micromotor in vivo and pH measurement by microelectrode pH meter. (**b**) The pH values in the stomachs of mice after different doses of Mg micromotor were given. (*n* = 3) (**c**) Twenty minutes after mice were injected with deionized water, Mg micromotors and inert polystyrene (PS) particles, superimposed fluorescent images of the whole stomach of mice were collected (both Mg micromotors and PS microparticles were loaded with DiD dye and encapsulated in a pH-sensitive polymer shell). Reproduced with permission from the publisher of corresponding reference [45].

As shown in Figure 2, Zhang et al. reported a novel magnesium-based micromotor covered by a pH-responsive polymer coating. Stomach acid was used as the motor, making it consume protons quickly as it propelled through the stomach, thereby increasing the pH in the stomach to neutral in less than 20 min, and this change allowed the cargo to be released from the pH-responsive polymer coating. Compared with the traditional pH passive responsive nanocarriers, this kind of micromotor could actively change the pH value of the surrounding local environment, so it could be used as a potential drug delivery carrier for the treatment of various gastric diseases [45].

Besides the development of drug delivery systems, pH-responsive systems can also be used for tumor detection and image-guided surgery [46]. By taking advantage of the difference of pH values in different target tissues in vivo, pH-responsive systems represent an intriguing strategy in drug delivery system. However, the difference of pH values between target and healthy tissues may not always differ significantly, so responsive systems that rely only on pH may be limited by low specificity and sensitivity. Therefore, pH responsive system can be combined with other stimulus conditions such as light, redox, enzymes and others with the aim of improved selectivity for drug release in diseased tissues [47,48].

### 2.2. Redox Stimulus

Redox conditions can be used for another type of stimulus-responsive system. These include GSH-sensitive systems, which have attracted much recent attention. GSH-sensitive systems can have stability in normal tissues but undergo release of cargo in diseased tissues in response to higher concentrations of GSH. For example, the concentration of GSH, as a tumor marker in cancer cells is 4–7 fold higher than in normal cells [49,50]. The intracellular concentration of GSH (1–10 mM) is about three orders of magnitude higher than that in the blood (2–10 µM) [51,52].

Disulfide bonds and diselenium bonds have been widely used in the design of redox stimulus responsive systems (Table 2). Disulfide bonds have also been used in chemical sensors. Shi et al. designed and synthesized a nanodrug carrier based on disulfide bond-doped organosilicon-micelle hybrid nanoparticles, the surface of which was modified with disulfide-bonded PEG and amide-bonded polyethylenimine (PEI). This nano-drug carrier exhibited excellent blood circulation ability and accumulation performance in tumor tissues, and the side effects of anticancer drugs on not-carcinogenic tissues were also reduced because of such rational design [53].

Compared with sulfur, the electronegativity of selenium is relatively low, and the atomic radius of selenium is larger, so selenium has some unique properties such as high reactivity and sensitivity (Table 2). The bond energy of S-S is 240 kJ·mol^−1^, whereas the bond energy of Se-Se bond is only 172 kJ·mol^−1^, suggesting that Se-Se bond may have higher sensitivity than disulfide bond; that is, Se–Se bond has the potential to release drug faster [54]. Wei et al. demonstrated that nanodrug carriers with diselenides could release more drugs using poly (ester carbamate) triblock copolymers (PAUR-Se-Se) with Se-Se bonds compared with poly (ester carbamate) triblock copolymers (PAUR-S-S) containing S-S bonds. At similar GSH concentrations (30 mM), 85% of encapsulated DOX was released by PAUR-Se-Se micelles within 48 h, while 67% of the drug was released by PAUR-S-S micelles. In addition, the antitumor effect of DOX-loaded PAUR-Se-Se was six fold higher than that of S-S analogues due to faster cleavage of the diselenium bond and enhanced drug release [55]. In a recent study, a new type of redox-responsive drug delivery system was developed based on the nano-metal-organic framework. Its core structure was formed by zirconium ions (Zr^4+^, metal nodes), 2,5-disulfanylterephthalic acid (BDC–(SH)_2_, organic ligand) and benzoic acid (BA, modulator), The sulfhydryl group on the anticancer drug, 6-mercaptopurine, formed a disulfide bond with BDC-(SH)_2_. This disulfide-based nanomaterial exhibited excellent intracellular drug release ability, and its cytotoxicity to cancer cells was shown to be three times higher than normal cells [67].

ROS-responsive systems are also an effective strategy to control drug release by utilizing the ROS accumulated in inflammation sites or other diseased tissues [68]. It has been shown that the concentration of ROS in inflammatory tissue is 10–100 times higher than that in normal tissue [69]. There are two chemical mechanisms commonly used in ROS-responsive systems. One is ROS-induced non-breaking hydrophobic-hydrophilic transition and the other is ROS-induced structural dissociation (Table 2). ROS can oxidize chalcogens (e.g., S, Se, Te) [56,57], causing oxygen atoms to form covalent bonds with chalcogens, and polarizing groups to form hydrogen bonds with environmental water molecules. Thus, hydrophobic-hydrophilic transition of the carrier backbone was induced without damaging the chemical structure of the drug carrier. Among the chalcogen elements, tellurium-containing compounds have more potential than other chalcogen elements due to their lower electronegativity and lower toxicity [58]. Additionally, it has been shown that tellurium-containing compounds have higher oxidation responsiveness than selenium-containing compounds and sulfur-containing compounds [59,60]. Xu et al. reported a synergistic therapeutic nanoplatform using near-infrared light-responsive cisplatin for cancer therapy. Cisplatin (CDDP) and indocyanine green (ICG) were simultaneously loaded in nanocarriers made of amphiphilic tellurium-containing block copolymer PEG-PUTe-PEG. Tellurium atoms in nanocarriers can be easily oxidized by ROS (stimulated by near-infrared laser) produced by indocyanine green. Tellurium oxidation weakens the coordination with cisplatin, thereby releasing cisplatin and achieving better anti-tumor effect [61].

**Figure 3:**
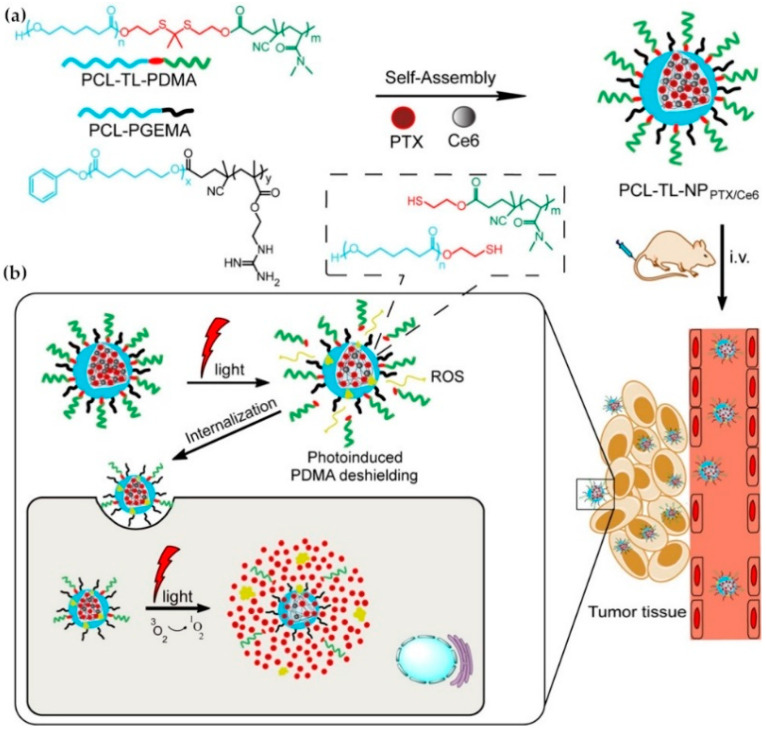
(**a**) Schematic illustration of preparation of ROS stimulus-responsive nanoparticles (PCL-TL-NP_PTX/Ce6_) loaded with Ce6 and PTX. (**b**) PDMA was cleaved upon the stimulation of ROS. Cell uptake under 660 nm light irradiation was improved. A synergistic effect of photodynamic therapy (PDT) and chemotherapy was achieved through efficient cell uptake and drug release. Reproduced with permission from the publisher of corresponding reference [73].

In addition, ROS can react with chemical structures such as thioketal (TK), phenylboronic acid/ester (PBA/PBE), oxalate ester [62,63], vinyldithioether [64] and proline oligomers, leading to the cleavage of these structures. In general, TK groups are easily oxidized to generate acetone and two other thiol-containing fragments in the presence of ROS. A novel amphiphilic peptide-drug conjugate for cancer targeted therapy has recently been reported. Hydrophilic cyclic peptides and hydrophobic cytotoxin epothilone B (Epo B) were linked by ROS responsive TK groups. When nanotherapeutics entered tumor cells, TK groups could be cleaved due to high levels of intracellular ROS, thereby releasing Epo B, which effectively inhibited cell proliferation [65]. H_2_O_2_ can specifically oxidize PBE/PBA and subsequently induce the formation of borate ester and hydroxybenzyl alcohol [66]. In addition, amino acids such as proline, histidine and arginine are also found to be susceptible to ROS-mediated and metal-catalyzed oxidation [70,71]. For example, Murthy et al. developed thioketal-based nanoparticles that could degrade selectively in the presence of ROS in inflamed intestinal tissue. In responsive to abnormally high levels of ROS in the sites of intestinal inflammation, thioketal nanoparticles could effectively deliver and release siRNA to diseased sites, protecting mice from dextran sodium sulphate (DSS)-induced colitis [72]. In addition to treating inflammation, ROS-responsive systems can also be used for cancer treatment. Ge et al. designed ROS-responsive nanoparticles made up by a thioketal linker (TL), poly (ε-caprolactone) and poly (*N*,*N*-dimethylacrylamide) (PCL-TL-PDMA). Amphiphilic di-block copolymers with PCL-b-poly (2-guanidinoethyl methacrylate) (PCL-PGEMA) were used to encapsulate the anticancer drug paclitaxel and a photosensitizer, achieving efficient cell uptake and enhanced anticancer efficacy [73] (Figure 3).

Redox stimulus-responsive systems need high sensitivity and accuracy for fast triggered responses in diseased tissues, but stability under normal conditions. However, considering the complexity and diversity of the microenvironment in vivo, delivery systems based on specific redox molecular mechanism that can release cargo in a controllable manner are preferred but not well established yet. Further investigations are expected to address these potential issues.

### 2.3. Enzyme Stimulus

Enzymes have attracted attention in various nanobiotechnology applications owing to their specific biological targeting and catalytic properties. In the microenvironment of the diseased sites, the levels of some enzymes can be abnormal; therefore, enzyme-responsive systems represent an appealing strategy for the development of responsive drug carriers. Enzymes can specifically target the affected sites, thereby regulating drug release. Hydrolases, including proteases, lipases and glycosidases, have been widely explored in enzyme-responsive systems. Oxidoreductases, such as NAD(P)H, quinone oxidoreductase isoenzyme I (NQO1) and glucose oxidase (GOx) have also been studied [74].

Proteases play important role in biological processes, such as differentiation, angiogenesis, hormone synthesis, digestion, wound repair, hemostasis, inflammation, coagulation, immune response, necrosis and apoptosis [75]. Some proteases are usually overexpressed in diseased tissues, and their activation can be used to release drugs for the development of protease-targeted therapies. For example, a new type of polymer poly (ethylene glycol) diacrylate (PEGDA) have been designed that could form spherical polymeric nanoparticles when mixed with peptide. The nanoparticles can be hydrolyzed by the overexpressed matrix metalloproteinase in lung lesions and then release the drugs [76]. In addition, it has been shown that the use of protease triggered drug release can enhance the therapeutic effect of drugs, and also reduce the toxicity and other side effects of drugs [77]. Cathepsin-B is normally expressed in the lysosomes of human cells, but is also overexpressed in many invasive and metastatic cancers. Therefore, cathepsin-B has been used as one of the targets of the enzyme-responsive systems. The amino acid sequence of Gly-Phe-Leu-Gly (GFLG) can be degraded in the presence of cathepsin-B, so it can be applied for the development of nanodrug carriers. For example, PEG, anticancer drug cisplatin and GFLG peptide were used to be assembled for cisplatin prodrug units. ICG was used to co-assemble with cathepsin-responsive cisplatin multidrug nanoplatforms. After cell uptake, the GFLG peptide structure was degraded by cathepsin B in lysosomes, which in turn released ICG and cisplatin prodrugs for cancer treatment [78].

Among many kinds of matrix metalloproteinases, MMP2 has been one of the most widely explored for stimulus-responsive systems because it is overexpressed in cancer tissues. MMP2-acting substrates have been used for drug delivery and molecular imaging. For example, Torchilin et al. synthesized an octapeptide (Gly-Pro-Leu-Gly-Ile-Ala-Gly-Gln) as a MMP2-sensitive linker for the conjugation of long-chain PEG to liposomes and encapsulation of the cell-penetrating peptide TATp. When octapeptide (GPLGIAGQ) was cleaved by MMP2, TATp was exposed, giving rise to the increased uptake of liposome particles by tumor cells [79]. In 2016, similar structures were also used in drug-loaded liposomes for pancreatic cancer treatment [80]. To improve permeability, antitumor efficacy and biodegradability, a size-shrinkable drug delivery system has been designed based on polysaccharide-modified dendrimers, which contained poly (amidoamine), hyaluronic acid and MMP2-sensitive peptide linker (PLGLAG). The system retained good stability in blood circulation, and underwent degradation upon the action of MMP2, leading to enhanced uptake in tumor cells [81]. In addition, polypeptide GPLGVRG [82,83], polypeptide GPLGVRGDG [84] and triglyceride monostearate (TGMS) [85] have also been synthesized for MMP2 sensitive drug delivery systems.

Phospholipase is another therapeutic target that can be overexpressed in infectious diseases, inflammatory diseases and tumors. In several inflammatory diseases or cancer cell types, the expression of phospholipase A2 (sPLA_2_) has been shown to be higher [86,87,88,89]. PLA_2_ is often used as a trigger condition in liposomal drug delivery systems, which specifically hydrolyzes sn-2 ester bonds in phospholipids, resulting in direct release of active drugs or hydrolysis of liposomes [90]. For example, colchicine-containing phosphatidylcholinase-responsive liposomes have been developed that are stable in human blood circulation. At the same time, liposomes could release colchicine-containing fatty acids upon the action of high levels of phospholipase A2. The latter further hydrolyzed and released colchicine analogues by non-specific enzymes [91]. Besides, in a recent report, an enzyme reactive liposome containing 1,2-dipalmitoyl-sn-glycerin-3-phosphocholine (DPPC) and 1-o-stearyl-2-retinoic acid receptor (RAR)-C6-sn-glycerin-3-phosphoglycerol with c6-RAR as a prodrug was designed. In the absence of sPLA_2_, the IC50 of c6-RAR prodrug in MT-3 breast cancer cell line was 110 μM, while the IC50 of c6-RAR prodrug decreased to 10 μm after adding sPLA2, indicating that the prodrug was hydrolyzed into c6-RAR and Lyso-O-SPG by sPLA_2_, in response to phospholipase [92].

Oxidoreductase has also been used as a potential target of stimulus-responsive systems due to its important role in intracellular oxidative environment. GOx is a kind of oxidoreductase tested for stimulus-response systems. It catalyzes the oxidation of β-d-glucose to d-glucose-δ- lactone and hydrogen peroxide with oxygen as electron acceptor [93,94]. Glucose responsive systems have been assessed for the treatment of diabetes [95,96]. For example, Willner et al. recently used zeolite imidazolium frameworks (ZIF-8 NMOFs) as a glucose-responsive carrier to release insulin and vascular endothelial growth factor (VEGF) aptamers, which inhibited angiogenesis by binding to VEGF proteins. ZIF-8 was stable under neutral physiological conditions but degraded under acidic conditions. Insulin or VEGF aptamers and GOx were integrated into ZIF-8 NMOFs as biocatalysts. GOx catalyzed the aerobic oxidation of glucose to produce gluconic acid. Local acidification of NMOFs degraded ZIF-8, thereby releasing insulin and VEGF aptamers [97]. Besides, NQO1 has been widely used in stimulus-responsive therapy systems due to its overexpression in tumors and other diseases [98,99]. In a recent report, a NQO1-responsive multifunctional polymeric vesicle covalently bound to a photosensitizer (coumarin and Nile blue) was prepared. Without being triggered by NQO1, both fluorescence emission and photodynamic therapy (PDT) capability were turned off due to the “double quenching” effect. Upon cellular uptake, highly expressed NQO1 triggered the self-immolative cleavage of the quinone trimethyl lock, which then led to the release of photosensitizers, near infrared (NIR) emission and PDT activation, thereby realizing real-time monitoring and treatment of cancers [100].

**Figure 4:**
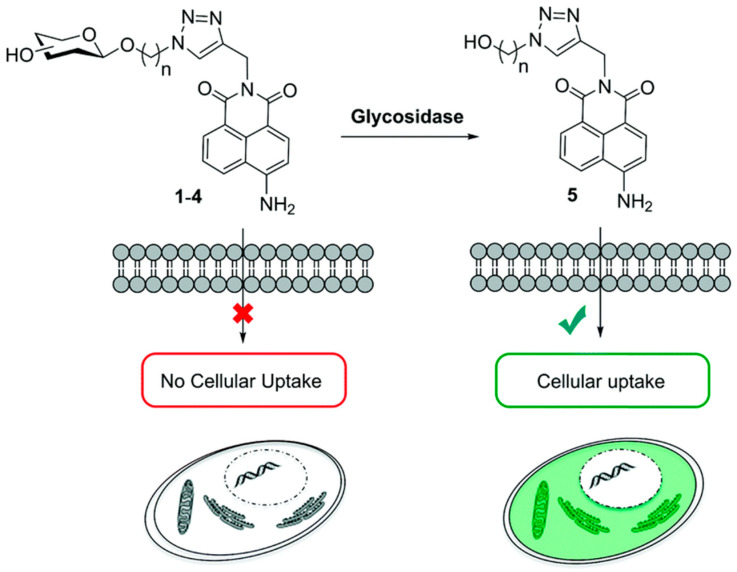
Glycosylated naphthalimide “probes” (**1**–**4**) are activated by glycosidases, resulting in the release of fluorescent probes (**5**), which can be endocytosed into cancer cells. Reproduced with permission from the publishers of corresponding reference [102].

Glucosidase is an enzyme that can hydrolyze glycosidic bonds. Its concentration in diseased tissues can be higher than that in normal tissues. Therefore, glucosidase can be used to design enzyme-responsive system. It was reported that the concentration of α-amylase could increase by 85-fold in the tumor environment [101], so it is an effective anticancer therapeutic approach to design glycosylated drug carriers to release anticancer drugs upon catalysis of glycosidase. For example, Scanlan et al. used glycosylated 1,8- naphthalimide as a fluorescent probe for tumor treatment and diagnosis. The glycosylated 4-amino-1,8-naphthalimide derivatives with chemical structures including the glycosidic bond can be selectively hydrolyzed by glycosidase to release naphthalimide. It was shown that naphthalimide was uptaken by cells only after the glycan unit was hydrolyzed, indicating high targeting capability of the delivery system [102] (Figure 4).

In spite of extensive development of enzyme-responsive systems, there are many drawbacks for them. The level of enzyme expression might be different in different patients, so whether they are sufficiently expressed in a target population is questionable. In addition, the specificity might be another issue. For example, various types of MMPs have cross-reactivity. Another consideration is whether the cleavage of the enzyme-responsive substrates and polymers is feasible in complex biological environment. The therapeutic effect of one single enzyme stimulus-responsive system might not be specific enough.

### 2.4. Light Stimulus

**Figure 5:**
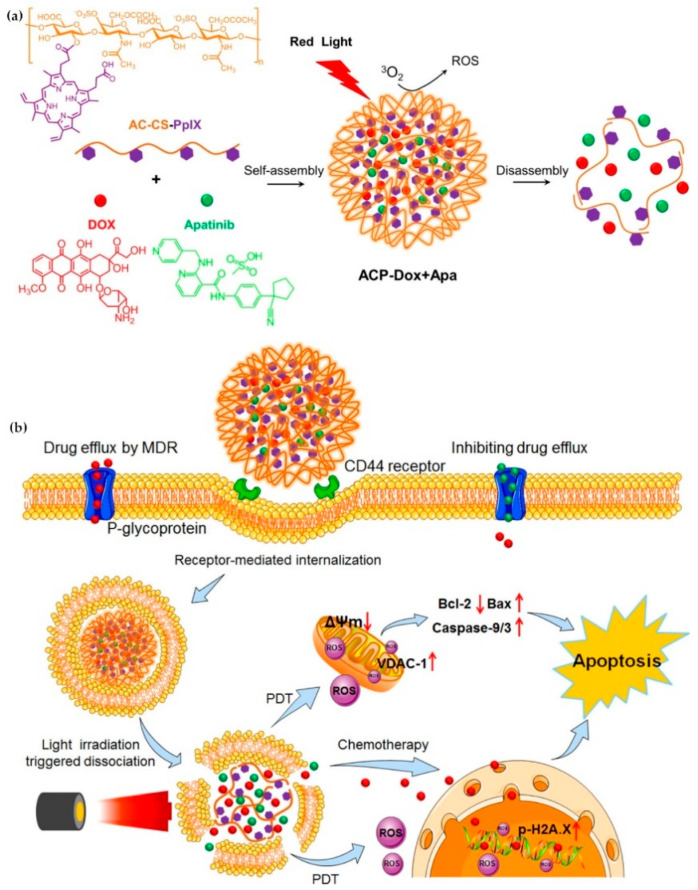
Schematic diagram of a light/ROS-responsive drug delivery platform. (**a**) Formation and cleavage of acetylated-chondroitin sulfate-protoporphyrin (ACP)-Dox + Apa micelles. (**b**) The mechanism of smart ACP-Dox + Apa micelles against multidrug resistance (MDR), for promoting the synergistic antitumor efficacy. Reproduced with permission from the publisher of corresponding reference [106].

Solid tumors have a variety of physiological barriers (such as high interstitial pressure and dense extracellular matrix) which affect the uptake of nanoparticles [103]. Photothermal therapy is a minimally-invasive treatment for cancer, which relies on the thermal stress caused by light irradiation at a specific wavelength [104,105]. PDT is another commonly used light-triggered strategy for disease treatments. Some examples of photosensitizers include Photofrin, Visudyne, chlorin e6 and oligo (p-phenylene vinylene) derivative (OPV). They can be activated by light to produce ROS, which can not only directly kill cancer cells, but also induce vascular damage, cause membrane oxidation and affect the permeability of cell membrane, which facilitates the penetration of anticancer drugs [32]. In a recent report, researchers designed a nano drug carrier that combines photosensitizer protoporphyrin, chemical drug doxorubicin (DOX) and apatite (APA). With light stimulation, DOX and APA were released and ROS was generated owing to porphyrins. APA competed with P-glycoprotein (P-gp) transporter to reduce its enzyme catalytic activity and DOX was used to treat tumor tissues; thus, the synergistic treatment of chemotherapy and phototherapy was achieved [106] (Figure 5).

In addition to photothermal therapy and PDT, light-responsive strategies have also been applied in the design of prodrug systems and drug delivery carriers. Light-responsive structure is generally a robust strategy because it is easy to control with good accuracy and minimal invasiveness. Light-responsive systems for drug release are often based on the cleavage of prodrugs with light-sensitive structures or the changes of photosensitive molecules upon light stimulation, so as to release the conjugated or encapsulated drugs [107,108]. Lovell et al. developed near-infrared light-responsive liposomes doped with hexyloxyethy-pyropheophorbide (HPPH) entrapped into the bilayers. The anticancer drug doxorubicin (DOX) was encapsulated inside the light-sensitive liposomes. When NIR light was irradiated at the target site, HPPH-liposomes opened the bilayer structure and then released DOX, but when NIR light was off, the stable bilayer structure of HPPH-liposomes was reformed [109]. Common photolytic functional groups include *O*-nitrobenzyl derivatives (ONB), anthracene [110], coumarin esters [111], arylmethyl [112] and pyrenylmethyl ester [113] (Table 3). Among these, *O*-nitrobenzyl derivatives have been widely used because of their fast photolysis rate and simple synthesis process. For example, Willner et al. designed a light-responsive and pH-responsive DNA microcapsule. Light-responsive capsules are assembled by *O*-nitrobenzyl phosphate groups with DNA layers used to stabilize the microcapsule shell (Table 3). When stimulated by light, *O*-nitrobenzyl groups were cleaved, causing the degradation of microcapsules and the release of the cargo [114]. Another report showed that the *O*-nitrobenzyl linker containing carbamate bond had a better photolysis rate and a slower hydrolysis rate than other *O*-nitrobenzyl derivatives (containing an ester bond or amide bond), so that it could maintain higher stability in vivo and can degrade faster after light stimulation [115]. Besides, Baker et al. chose ONB group as the core of photosensitive protective group and conjugated with poly (amidoamine) (PAMAM) dendrimer to develop a drug delivery platform for methotrexate (MTX) loading. It was demonstrated that this photochemical approach can be used for in vivo delivery of anticancer drugs [116]. Ultraviolet-Visible (UV-Vis) light could be used for the trigger of *O*-nitrobenzyl derivatives and aryl methyl photolysis functional groups; however, it is difficult to achieve tissue penetration for better therapeutic performance. Compared with UV and visible light, near-infrared light with wavelengths in the 700–1000 nm range can penetrate into deeper tissues. NIR could be used to cleave, isomerize or rearrange molecules through the two-photon absorption (TPA) process and NIR-UV upconversion (UC) process. The theoretical analysis on the principle of TPA process was first proposed in 1930s [117]. However, the generation of TPA requires high excitation energy, and there are still some problems in practical applications. In the UC process, the luminescence center can absorb the energy of two or more photons, and then generate one emitted photon whose energy is higher than that of a single excited photon. Additionally, unlike the TPA process, each process of photon absorption is like a second-order element reaction due to the existence of a real intermediate state in the UC process, which has a higher probability than the simultaneous absorption of two molecules [117,118]. Upconversion nanoparticles (UCNPs) based on rare earth ions provide a new method for the NIR light-responsive system. For example, Zou and his colleagues designed a near-infrared light-triggered nanocomposite PEG-NMAB-PLA-UCNPs (PNP-UC) to achieve photo-controllable release of DOX for cancer treatment. Under the irradiation of 980 nm NIR light, 2-nitrobenzyl group was triggered for its cleavage to release DOX. Compared with other UCNPs-based NIR responsive systems, this new nanocomposite has many advantages, such as high drug encapsulation efficiency, fast light responsiveness at low power density and less heating effect, showing potential in pharmaceutical and biomedical applications [119].

Common photoisomerization materials include azobenzene-based materials, spiropyran (SP)-based materials, and diarylethene-based materials (Table 3). Azobenzene is an azo group containing an aryl group, with cis and trans isomers. It shows a significant π-π transition in the UV region and a faint π-π transition in the visible region. Therefore, upon irradiation of UV light, the trans structure of azobenzene changes into cis structure. Additionally with heat or visible light, azobenzene can change from cis structure to trans structure [120]. Liu and colleagues assembled azobenzene-functionalized DNA strands using UCNP to construct nano-pumps and load DOX into nano-pumps. With 980 nm NIR irradiation, the continuous rotational inversion motion of azo molecules caused DNA hybridization and de-hybridization, thereby leading to the release of DOX. This novel light-responsive drug delivery system has shown good biocompatibility and excellent performance in both in vitro and in vivo therapies [121]. SPs are an important photoisomeric compounds. Under the UV light, the spiro-C-O bond in the spiropyran structure breaks open, leading to an increase in the degree of pi-electron conjugation of the whole molecular system, forming a long conjugate chain and the colored ring-opened merocyanine (MC). MC can be reversibly transformed into a colorless ring-closed SP under specific wavelength or heating conditions. Taking the advantage of this special property of SP, a novel *N*,*N*-bis(acryloyl) cystamine crosslinked poly (acrylic acid-co-spiropyran methacrylate) nanogel containing disulfide bonds was recently reported as a multi-stimulus responsive nanocarrier [122]. Upon the stimulation of low pH value or UV light, the hydrophobic SP isomerizes into hydrophilic MC, making the nanogel swollen. With the addition of reductant agent, the structure of nanogel was destroyed after disulfide bond was oxidized and cleaved, which caused the release of the loaded drug. Cytotoxicity experiments showed that DOX-loaded nanogels could effectively kill cancer cells, while their cytotoxicity could be enhanced by UV light irradiation. In addition, isomerized MCs in nanogels could emit strong green light after illumination, so they could also be used for fluorescent cell imaging.

Diarylethenes (DAEs) are also popular photoisomeric molecules with many useful properties, such as easy modification, fatigue resistance, high thermal stability and significant changes in optical and electronic properties after photoisomerization [123] (Table 3). The performance and application of diaryl ethylene mainly depend on the modification of its parental structure, that is, the design and selection process of alkene bridges and aryl units. One common way is the modification of aryl units such as substituting the benzene ring of diaryl ethylene with thiophene ring [124] (which could improve the stability of the closed loop) and modifying the alkene bridge using five-membered rings such as alkene bridges (such as cyclopentene [125], thiazole [126], imidazole [127], furan [128]) or using six-membered rings (such as benzoquinone [129], coumarin fluorophores [130], benzothiazole [131]). In addition, some light-sensitive molecules—vitamin B12 derivatives [132,133], ruthenium complexes [134], etc.—have also been applied to light-sensitive systems.

Still, further development of light-stimulated systems is hindered by some factors. For example, for the treatment of solid tumors, penetration depth, effective area, power intensity and irradiation time are some important factors to be considered for the light-responsive therapy. In addition, many photosensitizers and photothermal agents have inherent toxicity or photo-toxicity. Ongoing research is still making efforts for the application of light stimulation systems for clinical advances.

### 2.5. Temperature Stimulus

Temperature is a frequently studied stimuli for responsive delivery systems. Inflammatory pathological sites and the hyperthermic nature of tumors can be used as internal stimuli [135,136]. Another strategy is to increase the temperature by applying an external heat source. Ideally, the thermal response nanocarrier should maintain its stability at body temperature and release the drug rapidly when the diseased site is heated. Thermal stimulus-responsive polymers usually have lower critical solubility temperature (LCST) or upper critical solubility temperature (UCST), and the former is more widely used. For example, poly (*N*-isopropylacrylamide) (PNIPAM) and its derivatives have been used in the design of thermal stimulation response systems because their corresponding LCST is about 32 °C, which is close to the physiological temperature of human body [137,138,139].

A core-shell drug delivery system was designed to improve the solubility of drug by encapsulating hydrophobic drugs in nanoparticles with thermo-responsive materials as the shell. Luo et al. developed a core-shell micelle that used hydrophilic thermo-responsive material PNIPAM as a shell for the encapsulation of the drug of paclitaxel (PTX). Both drug and shell were conjugated by diselenide bond (PNIPAM-SeSe-PTX). The nanoparticles exhibited dual characteristics of temperature-responsiveness and redox-responsiveness, with high loading capacity (54.1%) and encapsulation efficiency (72.3%) [140].

Compared with PNIPAM that cannot be degraded in vivo, temperature-sensitive oligomers containing oligo (ethylene glycol) (OEG) potentially has better biocompatibility. Jayakannan et al. developed a dual stimulus-responsive amphiphilic copolymer formed by copolymerization of hydrophobic polymerizable monomers with methacrylamide based on hydrophobic 3-pentadecylphenol (PDP) and oligopolyethylene glycol (PEG) methacrylate, which thus was temperature sensitive and enzyme sensitive. The nanoparticles formed from the amphiphilic copolymer, which remained stable in normal tissues in vivo with less than 20% drug release, while above LCST, the nanoparticles had an up to 90% drug release rate in two hours due to the thermal response property of acrylamide copolymers. In the presence of esterase, the drug release rate could exceed 95% within 12 h. Such type of DOX-polymer showed better anticancer efficacy [141].

**Figure 6:**
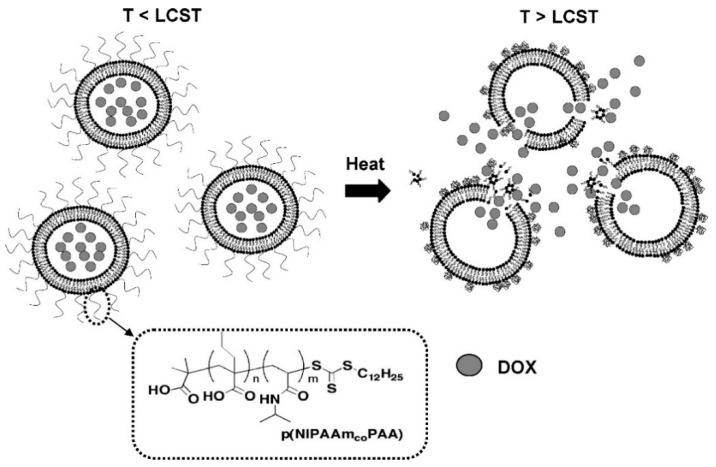
DOX release mechanism of polymer-modified thermosensitive liposomes. Heat caused the cleavage of the polymer chain and the degradation of bilayer structure, giving rise to the release of the encapsulated DOX. Reproduced with permission from the publisher of corresponding reference [142].

In addition to thermosensitive hydrogels and thermosensitive polymers, temperature-sensitive liposomes have also been investigated. Since Yatvin first proposed the concept of temperature-sensitive liposomes (TSL) in the late 1970s [8], TSL has been attracting much attention from molecular design to clinical applications. ThermoDox is an example of temperature-sensitive liposomes, which was tested in phase III clinical trials for hepatocellular carcinoma and phase II clinical trials for breast cancer. Its drug release rate is 5–50 fold higher than other liposomal drugs at elevated temperatures. Porter et al. developed a novel polymer-modified temperature-sensitive liposome (pTSL) for the delivery of DOX for the treatment of cancers. By reversible addition-break chain transfer (RAFT) polymerization of *N*-isopropylacrylamide (NIPAAm) and pH-responsive propylacrylic acid (PAA) copolymers with temperature responsiveness, copolymers with dual pH/temperature phase transition properties were obtained. When attached to liposomes, these copolymers can cause membrane destruction with a pH/temperature-dependent manner. Compared with traditional thermosensitive formulations, pTSL exhibited enhanced release profiles, significantly reduced thermal dose thresholds and better stability in serum with minimal drug leakage over time. Therefore, these liposomes have the potential of significantly reducing damage to healthy tissue commonly associated with liposomal cancer therapies [142] (Figure 6).

For temperature-sensitive systems, thermo-specificity is one of the biggest issues because it is usually hard for chemistry to be exquisitely specific. For the future research directions, temperature-responsive drug carriers with higher sensitivity to the minimum temperature change, better stability and enhanced safety profile in normal tissues are greatly desired.

### 2.6. Magnetic and Ultrasound Stimulus

**Figure 7:**
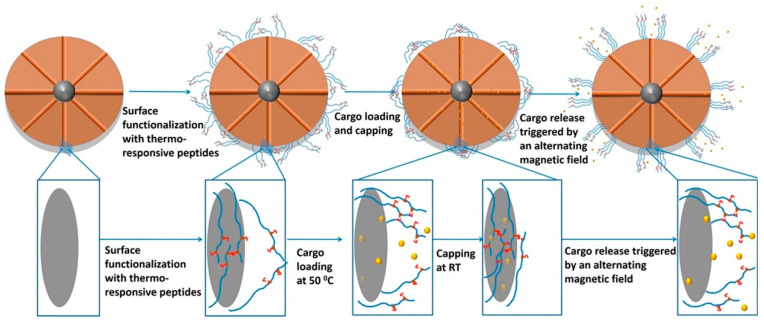
Schematic diagram of the working principle of heat-responsive peptide-modified nanovalve. The heat caused the decomposition of the peptide, leading to the release of the cargo from the pores of the MSN. Reproduced with permission from the publishers of corresponding reference [146].

Compared with other stimuli such as light radiation and ultrasonic, magnetic force is an intriguing condition for external stimuli-responsiveness because it has almost no physical interaction with the human body. Additionally, owing to spatial focusing, magnetic stimulus-responsive systems can overcome some limitations of traditional delivery systems, such as difficulty of passing through physiological barrier in vivo and lack of specificity to diseased tissues. Magnetic fields was first proposed as external triggers for drug release in 1960 [143]. Magnetic hyperthermia caused by the rotation of magnetic nanoparticles when exposed to alternating magnetic field, and the heat generated by magnetic loss will dissipate to the surrounding tissues. It is generally believed that the heat generated by magnetic nanoparticles is based on mechanisms of internal rotation axis and external movement, namely, thermal rotation and diffusion relaxation of magnetic moment. In terms of local drug release, the thermodynamic phase and conformation transition of polymeric nanoparticles depends on their LCST/UCST and then expands/contracts, possibly leading to the drug release [144,145]. Zink et al. developed a magnetic nanoparticle with superparamagnetic particles as the core and thermo-responsive peptide Phe-Phe-glycine-glycine (*n*-porcine linolenic acid a) as the nano-valve. This novel class of Mesoporous silica nanoparticles (MSNs) could maintain stability and safety under normal environment in human body. Once the magnetic core is triggered by magnetic field, the heat generated can lead to the change of its structure, giving rise to the release of drugs [146] (Figure 7).

Magnetic transfection or magnetically-aided transfection is a method to control gene delivery and improve transfection efficiency for gene therapy. The magnetic transfection method is based on the principle of magnetic targeted drug delivery proposed by Widder et al. in 1978 [147]. In 2002, Mah et al. first linked the magnetic microspheres to recombinant adeno-associated virus 2 (AAV2) gene vector through heparin for C12S cells and mice in vivo [148]. In the same year, Plank et al. associated gene carriers with superparamagnetic iron oxide nanoparticles coated with polycation polyethyleneimine. It was shown that the magnetic-assisted transfection method improved the transfection efficiency of vectors by at least three orders of magnitude by either viral or non-viral vectors [149].

Ultrasound is also commonly used in stimulus responsive system. Compared with other external stimuli, ultrasound is non-invasive and can improve drug release profile and drug permeability. The mechanism is based on acoustic cavitation, which means the formation and activity of gas-filled bubbles in the medium under the action of ultrasound [150]. These gas-filled bubbles can be produced either naturally or manually (microbubbles, MBs). With microbubbles, ultrasound cavitation affects the permeability of the plasma membrane, enhances cell uptake of drugs, or allows internalization of other cell-impermeable substances. Even in the absence of microbubbles, high-intensity ultrasound can still induce drug uptake by cells. These characteristics enable ultrasound-mediated drug delivery systems to improve the absorption efficiency of weak or non-permeable drugs by cells [151]. Porous lipid polymer hybrid microbubbles (lipid/PLGA MBs) have also been developed using water/oil/water (W/O/W) double emulsification process, which addressed the limitation of low drug encapsulation efficiency of lipid-based MBs and poor ultrasound imaging ability of polymer-based MBs. Compared with pure PLGA MBs, lipid/PLGA MBs had hollow microcapsule structure, which reduced the cavitation threshold intensity and enhanced the ultrasonic imaging ability of MBs. In addition, the increased surface area and porous structure of lipid/PLGA MBs make them have good drug encapsulation properties. Using this method, controllable drug release and real-time monitoring of drug release could be achieved [152]. Another example is that Zheng et al. developed ultrasound stimulus-responsive microbubbles for ultrasound imaging, which can be converted into porphyrin nanomaterials with fluorescent and photoacoustic activity upon low-frequency ultrasound pulses. Larger microcarriers could reach the tumor vasculature (independent of EPR effect), and its principle is that the energy of ultrasound could rupture microbubbles, pushing nanomaterials into the tumor interstitium. These nanomaterials (porphyrins) could further utilize photoacoustic imaging (PAI) and fluorescence for multistep multimodal imaging in tumor-bearing mice [153].

**Figure 8:**
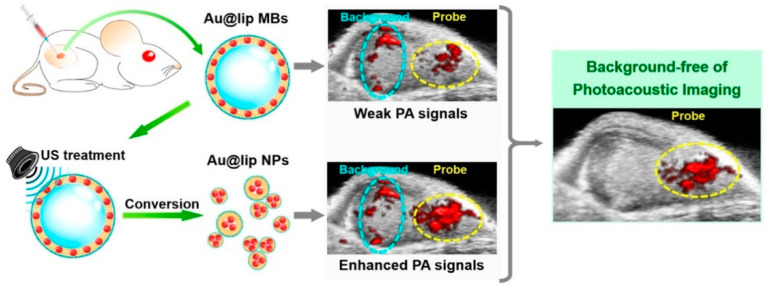
Schematic illustration of the ultrasound-responsive Au@lip MBs used for in vivo photoacoustic imaging with minimal background. Reproduced with permission from the publisher of corresponding reference [154].

Liu et al. recently designed an ultrasound responsive photoacoustic (PA) probe based on microbubbles containing gold nanoparticles. In this design, gold nanoparticles were encapsulated in the lipid shell of MBs and filled with sulfur hexafluoride gas, thereby forming MBs with strong cavitation characteristics and low toxicity. Au@lip MBs exhibited lower NIR PA signal. When triggered by ultrasound, MBs was ruptured and Au@lip aggregates were formed, showing enhanced NIR PA signals. Background-free PA imaging was achieved by subtracting the PA image before US stimulation from the PA image after US stimulation [154] (Figure 8). Although ultrasound-responsive nanotechnology shows some advantages, its side effects (such as skin irritations, transient pain and nerve injury) should be also considered [155,156]. The intensity, frequency and duty cycle should be carefully selected for the optimized therapeutic effects and reduced side effects.

### 2.7. Self-Immolative Structures

**Figure 9:**
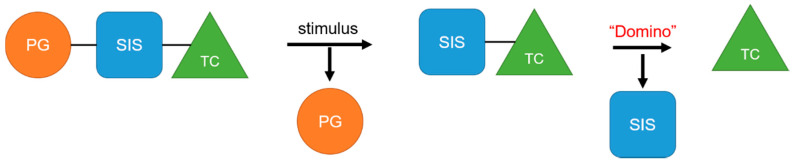
Schematic representation of a self-immolative spacer. PG, SIS and TC stand for protection group, self-immolation spacer and target compound, respectively.

The mechanism of self-immolation is that two chemical bonds in the inactive precursor are associated by the self-immolation spacer. The precursor usually contains a protective group (PG), self-immolation spacer (SIS) and a target compound (TC). After appropriate stimulus, the protective group is removed, and then a step-by-step decomposition reaction similar to “domino” is produced, resulting in the release of target compounds (Figure 9). Philip et al. Proposed the concept of self-immolative structures in 1981 that a pristine drug can be released without chemical modification after the bond between the carrier component and the drug component is cleaved [157]. Self-immolative linkers have subsequently been widely used in prodrug design [158,159], sensors [160,161], drug delivery [162,163] and other applications.

The self-immolation mechanism can be categorized into electron rearrangement and intramolecular cyclization. Electron rearrangement is mainly based on the structure of quinone or its derivatives (such as thioquinone methide or azaquinone methide). The mechanism of electron rearrangement includes 1,4-elimination, 1,6-elimination, 1,8-elimination and β-elimination. The electron rearrangement reaction usually contain aromatic ring structure with hydroxyl [164,165], amino [166,167] or mercaptan groups [168,169]. When they are masked by protective groups, the electron rearrangement process of these functional groups is inhibited. When the external stimulus triggers the cleavage of the protective groups, these functional groups undergo irreversible self-immolation process driven by positive entropy or the generation of stable products. Common protection groups that can be triggered by various stimuli are summarized in Table 4. Self-immolative spacers based on electron rearrangement are mostly based on 1,4-elimination or 1,6-elimination [170,171]. Additionally, 1,8-cleavage may occur in para amino (or hydroxy) cinnamyl alcohol or coumarin alcohol. In contrast, self-immolative spacer groups based on continuous combinations (such as 1,8-elimination of naphthalene or 1,10-elimination of biphenyls) usually do not result in the release of drug groups because the high energy barrier destroys the aromaticity, and the repulsion of the hydrogen atom in adjacent biphenyl prevents the formation of the planar structure needed for electron rearrangement [172]. The cyclization reaction is based on alkyl chain or aromatic spacer groups [173,174,175]. Once the cleavage is triggered, the nucleophilic attack of carbonyl or electrophilic aliphatic carbon atoms could result in the cyclization of spacer groups. As of the electron rearrangement, the self-immolation cyclization is driven by the formation of positive reaction entropy and thermodynamic stable products (such as 5-membered and 6-membered rings). Additionally, the triggers of self-immolation include chemical reagents, enzymes, light and others.

Self-immolative linkers that can be triggered by chemical reagents; Boc(Tert-butoxycarbonyl), Fmoc(9-fluorenylmethyl) and 3-oxobutyl carbamate are typically pH-responsive for organic synthesis [176]. Boc and Fmoc are protecting groups for protecting amino groups, and the Fmoc is the only widely used amino acid protecting group of carbamates which can be dissociated in weak base condition. These functional groups undergo self-immolation under some specific triggering conditions (trifluoroacetic acid, piperidine), which in turn releases the attached cargo [177,178]. Redox-triggered self-immolative linkers could be broadly divided into three categories by triggers: transition metal (e.g., Zn, Pd) based reagents [179,180,181], reducing reagents (DTT, GSH or TCEP) [182], and oxidant (e.g., H_2_O_2_) [183,184,185,186]. Disulfide bonds are the most widely used reductant-responsive linkers. Wu et al. employed α, α-dimethyl groups with disulfide bonds, so that p-dithiobenzyl (DTB) intermediates could maintain faster self-immolation rate and improve the stability. This novel self-immolative linker is expected to promote the design of targeted drug delivery systems and achieve traceless drug release [187]. Similarly, self-immolative linkers with H_2_O_2_ as trigger condition have also been widely used in stimulus-responsive systems in various fields. Recently, Clausen et al. reported the synthesis of arylboronic acid-based hydrogen peroxide-responsive methotrexate and aminopterin prodrugs. This new prodrug can deliver drugs to the lesion of chronic rheumatoid arthritis, so that the side effects of the drug on normal cells were significantly reduced [186]. Enzyme sensitive self-immolative linkers are also commonly used in enzyme responsive systems. Plasmin can induce the hydrolysis of tripeptides, which in turn causes the cleavage of self-immolative linkers and release of drugs [172,188]. Penicillin G amidase (PGA) and bovine serum albumin (BSA) can be used to trigger the cleavage of phenylacetamide or carbamate bonds to release the linked cargo [189,190]. β-galactoside and β-glucuronide are functional groups that can be cleaved by β-galactosidase and β-glucuronidase, respectively, and these two functional groups can also be combined with other self-immolative linkers [191,192,193,194]. In addition, dibenzyl phosphate derivatives are also used for enzymatic (alkaline phosphatase) self-immolative design for the delivery and release of fluorescent probes and prodrugs [195,196]. Enzymatically activated self-immolation is usually slow and requires synergy with other self-immolative linkers. Light-activated self-immolation does not require an additional step for synergy. A variety of similar structures based on nitrobenzyl groups can undergo self-immolation under UV light [197,198]. Examples of self-immolative structure triggered by NIR light are polymers with o-nitrobenzyl group as the capping group [199,200] and polymers based on coumarin derivatives [201,202].

## 3. Applications of Stimulus-Responsive Systems

### 3.1. Therapeutic Applications

Conventional drug delivery systems lack controlled drug release schemes whereas stimulus-responsive drug delivery systems or drug conjugates with rational design can release loaded drugs at specific sites triggered by various endogenous or exogenous stimuli. The human body is a complex collection of various microenvironments, so occasionally it may not suffice if only one stimulation condition is used for the design of stimulus-responsive systems. In this section, we mainly describe the combination of dual or multiple stimuli-responsive conditions for the design of nanoplatforms to improve the specificity and accuracy of nanotherapeutic systems.

#### 3.1.1. Cancers

**Figure 10:**
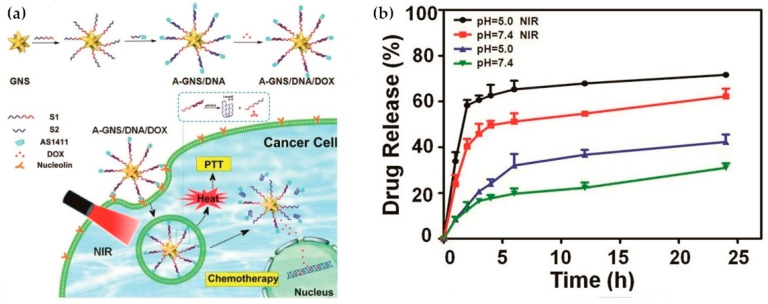
(**a**) Schematic illustration of NIR/pH dual stimuli-responsive nanodrug carrier (A-GNS/DNA/DOX) for chemical/PTT combination therapy for cancer. (**b**) Cumulative DOX release from A-GNS/DNA/DOX in the presence or absence of NIR and at different pH values. Reproduced with permission from the publisher of corresponding reference [206].

Although single stimulus-responsive systems can solve the problems of specificity and side effects to some extent, the microenvironment within tumor tissues is complex. By contrast, dual or multiple stimuli-responsive nanocarriers can better detect subtle changes in diseased tissues. The traditional combination of pH and temperature has certain defects, such as possible premature drug release in blood or normal tissue species [203,204], and the pH responsive system might be affected by various factors. To solve the problem of premature leakage, Cui et al. made *N*-isopropylacrylamide-methacrylic acid-octadecyl acrylate (NIPAM-MAA-ODA) copolymer liposomes as nanodrug carriers that can be responsive to dual stimulates of pH and temperature [205]. In recent years, the combination of light and pH has also become a popular choice for dual response systems. For example, researchers have conjugated pH-sensitive I-motif DNA (converted from single-stranded structure to C-quadruplex structure in acidic pH environment) to gold nanostars (GNS) with AS_1411_ used as a targeting structure. It has been discovered that A-GNS/DNA/DOX nanocomposites have strong photothermal conversion ability, and the combination of pH and NIR irradiation can effectively trigger drug release. This nanocomposite showed good stability in not-carcinogenic tissues, and therapeutic effect and biocompatibility were achieved using the construct for combined chemo- and photo-therapy [206] (Figure 10). In addition to the combination with photothermal therapy, pH responsive systems can also be combined with the UV light-responsive systems. Host-guest interaction of β-cyclodextrin with azobenzene has also been studied, but most supramolecular polymer drug carriers are single stimulus-responsive with low delivery accuracy [207,208,209]. Chen et al. recently constructed supramolecular polymers harnessing host-guest interaction between β-cyclodextrin and azobenzene. β-cyclodextrin was combined with pH-sensitive hydrophilic poly(2-(dimethylamino) ethyl methacrylate, and azobenzene was modified with hydrophobic poly(ε-caprolactone), enabling nanodrug micelles to be responsive to both pH and UV light. Additionally, higher anticancer activity and stronger cancer cell inhibition than free DOX could be achieved using this method [210]. Dual stimuli-responsive system using pH and redox is also a common combination. With low pH and high GSH levels in tumor cells, a strong controlled release effect can be achieved. Recently, pH/redox-responsive mixed polymeric micelles formed by self-assembly of two amphiphilic diblock copolymers (poly(ethylene glycol) methyl ether-b-poly(β-amino ester)) (mPEG-b-PAE) and poly(ethylene glycol) methyl ether-grafted disulfide bond-poly(β-amino ester) (PAE-ss-mPEG) have been developed, which released drugs at low pH and high GSH concentration in tumor cells [211]. In addition, there are also many combinations, such as pH/ROS responsive system [212,213], pH/enzyme responsive system [214,215] and others. As a significant characteristic stimulus condition in vivo, the combination of pH with other stimulus has shown promise for cancer treatments.

**Figure 11:**
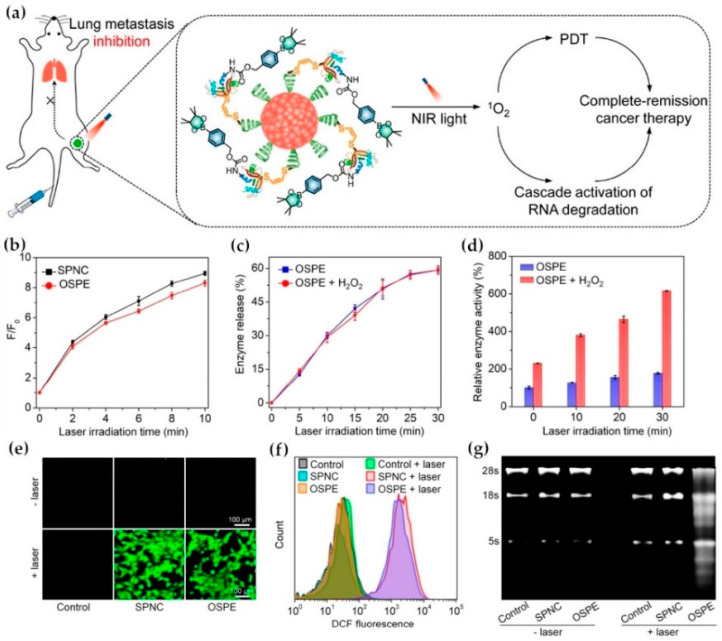
(**a**) Anticancer mechanism of NIR/^1^O_2_ dual stimuli-responsive organic semiconductor pro-nanoenzyme (OSPE). (**b**) Under NIR irradiation, ^1^O_2_ was generated by SPNC (control nanoparticles without enzyme modification) or OSPE. (**c**) The correlation of the amount of enzyme released from OSPE with the time of NIR irradiation. (**d**) In the absence and presence of H_2_O_2_, the relative enzyme activity of OSPE after NIR light irradiation for different times. (**e**) Confocal fluorescence images and (**f**) flow cytometry analysis of 4T1 cancer cells treated with SPNC or OSPE ([SPN] = 40 μg/mL) with or without 808 nm laser irradiation (0.3 W/cm^2^) for 10 min. (**g**) The agarose gel electrophoresis of RNA extracted from 4T1 cancer cells, treated with SPNC or OSPE ([SPN] = 40 μg/mL), and treated with or without 808 nm laser irradiation (0.3 W/cm^2^) for 10 min. Reproduced with permission from the publisher of corresponding reference [223].

Redox and ROS responsive systems are often used in combination with exogenous stimuli to achieve better stability and controlled release schemes. These two stimuli conditions can be combined to form a dual stimuli-responsive systems [216]. For example, Xu et al. copolymerized two camptothecin (CPT) prodrug monomers with disulfide bond and oxalate bond on β-cyclodextrin and hydrophilic poly (ethylene glycol) methyl ether methacrylate (OEGMA) to prepare a novel dual redox stimulus-responsive prodrug. This prodrug has small particle size, high stability, excellent biocompatibility, good permeability, improved safety and high anti-tumor efficiency. This prodrug strategy is expected to provide a feasible method for advanced chemotherapy [217]. In addition, redox/light responsive system is also an effective strategy for cancer treatments due to the scheme of dual triggers [218,219]. In a recent report, a thioacetal-based ROS-sensitive amphiphilic copolymer (PTK) was developed to load NIR cyanine dye IR 780 and DOX to obtain stable nanoparticles (IR780/DOX@PTK). IR 780 was used as photosensitizer, photothermal agent and imaging contrast agent simultaneously [220,221]. Upon the irradiation of 808 nm NIR light, IR 780 could not only initiate PDT and PTT, but also used for photothermal imaging. The generated singlet oxygen (^1^O_2_) led to the cleavage of thioacetal linker in PTK and the release of DOX, enabling chemotherapy and phototherapy [222]. Pu et al. synthesized an organic semiconducting pro-nanoenzyme (OSPE) recently, which could be activated by NIR light irradiation. OSPE is made by binding semiconductor polymer nanoparticles (SPN) to proenzyme (based on cytotoxic ribonuclease A) via singlet oxygen (^1^O_2_)-sensitive linkers. When SPN released singlet oxygen, it could not only carry out photodynamic therapy, but also released proenzyme to degrade cancer-specific RNA, the synergistic treatment by chemo/phototherapy for cancers [223] (Figure 11). Other dual/multiple stimuli-responsive systems for cancer treatments include pH/magnetic responsive systems [224,225] and other pH-based multiple stimuli-responsive systems [226,227,228].

#### 3.1.2. Inflammation

Inflammation is an immune response to infection and tissue damage so that the body can be protected from injury. However, it can also cause many diseases such as asthma, cardiovascular diseases, neurodegenerative diseases and autoimmune diseases (including rheumatoid arthritis, systemic lupus erythematosus and other diseases) [229,230]. These inflammatory chronic diseases can seriously affect health, and there are many obstacles to the treatment of the inflammatory chronic diseases.

The characteristic conditions within the microenvironment of inflammatory tissues include lower pH value and higher ROS concentration [231,232]. Therefore, pH/ROS dual stimulus-responsive system is the most widely used treatment strategy for inflammation therapy [233,234]. For example, Almutairi et al. designed a ROS-reactive dextran-drug conjugate (Nap-Dex) and blended Nap-Dex with an acid-sensitive acetal-dextran polymer (Ac-DEX) to obtain pH/ROS dual stimulus-responsive nanoparticles. The ROS-responsive PBA-modified anti-inflammatory drug naproxen was used. When the nanodrug was stimulated by H_2_O_2_ and acidic environment, the Ac-DEX and PBA structure of the nanodrug micelle was cleaved, thereby releasing naproxen (Nap) for the treatment of inflammatory tissues. Dual stimuli-responsive nanodrug micelles are more effective in scavenging ROS. Compared with free naproxen, dual stimuli-responsive nanoparticles reduced the levels of proinflammatory cytokines IL-6 and TNFα by 120 times and 6 times respectively [235]. In addition to lower pH and higher ROS concentrations, inflammation of tissues tends to have slightly increased local temperature (1–2 °C above ambient temperature) [236]. Therefore, the temperature/pH dual stimulus-responsive system has also been applied in the treatment of inflammation [237,238]. A microbead system was designed with a porous poly (D, L-lactic-co-glycolic acid) (PLGA) shell coupled with a gelatin plug (thermal-responsive switch). Additionally, *N*-palmitoyl chitosan (NPCS) (pH-responsive switch) was employed as a core. Vancomycin was loaded in the nanoparticles as the inflammatory therapeutic drug. This dual stimuli-responsive drug MBs could release the drug only in the presence of both stimuli (temperature and pH), preventing unexpected drug release due to accidental stimulus [239].

**Figure 12:**
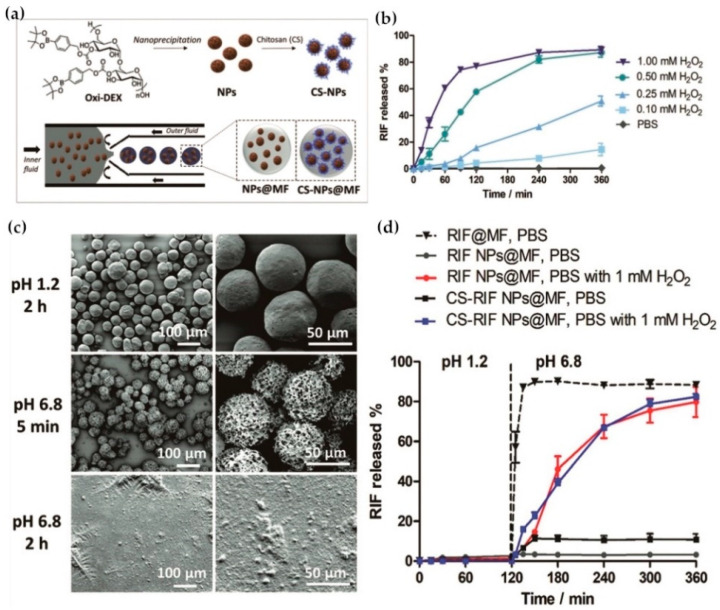
(**a**) The synthesis strategy of NP and CS-NP, and the schematic diagram of the preparation of nanocomposites. (**b**) OxiDEX NPs released Rifaximin (RIF) in PBS buffer solutions with different concentrations of H_2_O_2_. (**c**) The SEM images of RIF NPs@MF that were dissolved in simulated gastric fluid (SGF) (pH 1.2) for 2 h, and in PBS solution (pH 6.8) for 5 min and 2 h. (**d**) Profiles of drug release from nanocomposites (RIF NPs@MF and CS-RIF NPs@MF) and from RIF@MF formulations. Samples were first incubated in SGF (pH 1.2) for 2 h and then in PBS solution (pH 6.8) with or without H_2_O_2_ for 6 h. Reproduced with permission from the publisher of corresponding reference [243].

Inflammatory bowel disease (IBD) is a chronic condition of idiopathic inflammation. IBD can affect the entire gastrointestinal tract (GI) and increases the risk of colorectal cancer [240]. The incidence of IBD worldwide has increased and effective treatment approaches are needed. Therefore, drug delivery systems utilizing IBD microenvironment characteristic conditions (low pH, colonic enzymes, and high ROS concentration) as responsive factors have attracted extensive attention [241,242]. Li et al. reported a nano-platform of oxidatively sensitive dextran (OxiDEX) with its exterior modified by chitosan (CS) and then further encapsulated by pH-sensitive hydroxypropyl methylcellulose acetate succinate (HPMCAS). The intestinal-specific antibiotic rifampicin was used as a model drug to form pH/ROS dual stimulus-responsive nanodrug composites. This novel nanodrug composite remained stable in the upper gastrointestinal tract, but HPMCAS were cleaved in the intestine, thereby releasing nanodrug particles. Triggered by higher ROS levels, rifampicin is released into inflamed tissues. Compared with traditional enteric drug formulations, this nanodrug composite effectively reduced the permeability of drugs at the intestinal epithelium, preventing non-specific absorption and side effects [243] (Figure 12). In addition, some oral drug formulations responsive to pH have been used for clinical treatments. Examples include 5-aminosalicylic acid encapsulated in capsules made from copolymers of acrylic acid derivatives and methyl methacrylate (Eudragit^®^), such as Salofalk^®^, Calitoflak^®^, Claversal^®^, Pentasa^®^ and other brands [244,245,246].

Other inflammation examples include neuroinflammation, which represents an abnormal condition of central nervous system in many neurodegenerative diseases [247]. Some serious neurodegenerative diseases include Alzheimer’s disease, epilepsy, Huntington’s disease and Parkinson’s disease [248,249,250]. Strategies for the treatment of these diseases are desired and some ongoing efforts have been made on stimulus-responsive systems that can target excessive ROS concentrations in neuroinflammatory tissues [251,252].

#### 3.1.3. Oral Medications

Oral medication is the preferred route of administration because of better patient compliance. However, many drugs themselves (protein drugs/polypeptide drugs) are sensitive to the acidic and proteolytic environment of the gastrointestinal tract, so oral administration remains a challenge. Factors for oral administration that should be considered include (1) the activity of biopharmaceutical macromolecules in the gastrointestinal (GIT) environment, (2) the permeability in the intestine, and (3) drug molecules can be absorbed into the systemic circulation through the intestine [30,253].

Diabetes mellitus is an endocrine disease characterized by hyperglycemia, which is difficult to cure and can lead to various serious complications. The conventional way to treat diabetes is to inject insulin using subcutaneous administration, but the development of an effective orally administered insulin formulation would be preferred. The first attempt to treat diabetes with oral insulin can date back to 1922 but the failure made researchers realize that the main obstacle to oral delivery of biomacromolecules was mainly from the human body itself [254]. Chitosan/insulin/heparin sodium (CS/Ins/HS) nanoparticles were synthesized by ionic gel method, in which heparin sodium with three acidic functional groups can enhance the stability of the nanoparticle system in the stomach. Mucosal affinity for CS/Ins/HS in the small intestine were also improved due to the interaction of ins/HS with the positive charge on chitosan. In addition, Acrylate-grafted-carboxymethyl starch (CMS-g-AA) and methacrylic acid (MAA) were used to synthesize pH/amylase dual stimuli-responsive hydrogels (CMS-g-AA/PMAA). Drug-loaded hydrogels can be contracted in the stomach to ensure the stability of nanoparticles. However, in the small intestine the hydrogel can be swollen so that insulin could be released, resulting from the carrier degradation by intestinal amylase. Additionally, because of chitosan and heparin sodium, the reversible opening of the pathway between adjacent intestinal epithelial cells allowed insulin molecules to pass through the intestinal epithelial cells. This approach can also be used for the design of orally administered insulin formulations [255].

**Figure 13:**
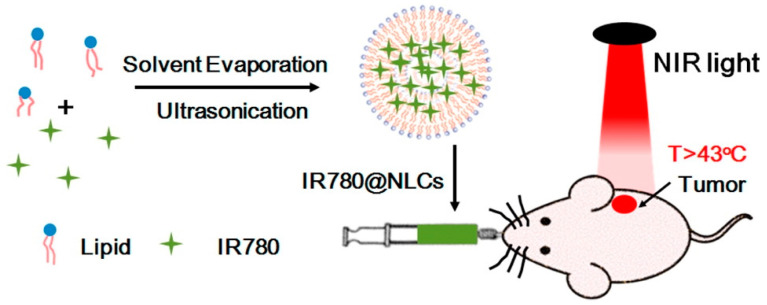
Preparation of IR780@NLC and the mechanism of anticancer therapy. Reproduced with permission from the publisher of corresponding reference [260].

In addition to oral insulin, many stimulus-responsive systems have been designed for the treatment of other diseases such as Helicobacter pylori [256], various cancers [257,258,259]. For example, Oupciky et al. reported a nanostructured lipid carrier (NLC)-based PTT formulation that can be orally administered. NLC showed its excellent biocompatibility, degradability and stability in the gastrointestinal tract. Under NIR light, NLC loaded with IR780 can be used for photothermal treatment on cancer cells [260] (Figure 13). Zhang et al. developed a supramolecular elastomer gel based on poly (acryloyl 6-aminocaproicacid) (PA6ACA) and poly (methacrylic acid-ethyl acrylate) (EUDRAGIT L 100-55), which remained stable in acidic gastric environment, but dissolved in the small intestine with neutral pH, allowing safe passage through the stomach and into the intestine afterwards. Under acidic conditions, carboxyl groups were protonated, and the inter-chain hydrogen bonds between carboxyl groups and amide units on PA6ACA and L 100-55 formed a loosely cross-linked supramolecular network with water trapped inside, providing good elasticity and stability. Under neutral pH conditions, the carboxyl groups in the molecule underwent deprotonation, and the disappearance of hydrogen bonds made the gel dissolve quickly. This type of gel material could be used as a good carrier for oral drugs with the advantages of strong elasticity, easy compression and folding. The use of this material for oral delivery drug was also evaluated in pigs [261]. Stimulus-responsive oral drug delivery systems are still under development and much efforts are being endeavored in order to improve delivery efficiency and targeting capability and to reduce the side effects (e.g., potential pathogenic risk of agents used for increased intestinal permeability).

### 3.2. Applications for Bio-Imaging

Non-invasive bioimaging approaches have been used for observation of biological activities and disease diagnosis. Commonly used bioimaging techniques include optical imaging, magnetic resonance imaging (MRI), ultrasound (US), computed tomography (CT), positron emission tomography (PET). Photoacoustic imaging is another emerging imaging modality that shows potential for future clinical use. In this section, we will mainly introduce the latest research results of stimulus responsive nano-systems used for bio-imaging.

**Figure 14:**
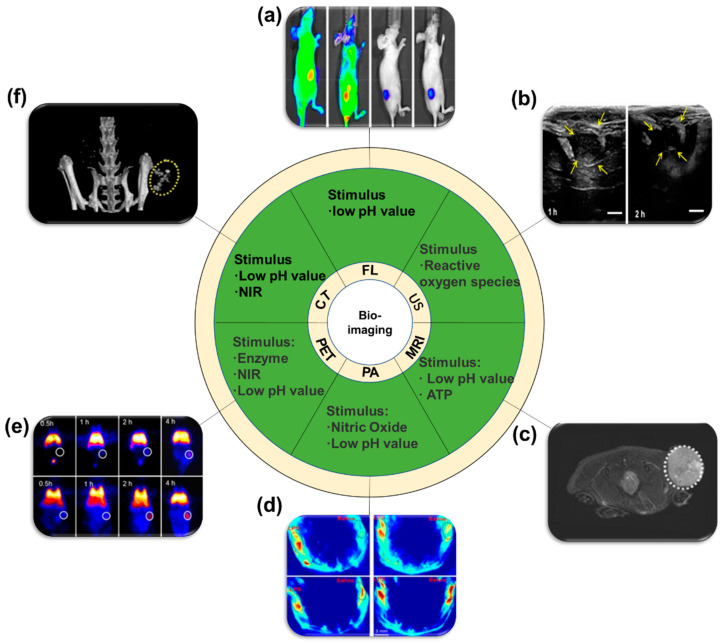
Stimulus-responsive systems for different bioimaging modalities. (**a**) Responsive to the acidic microenvironment in tumors, nanoprobes significantly enhanced tumor fluorescence imaging [265]. (**b**) ROS significantly enhanced the ultrasound signal [266]. (**c**) MR contrast could be enhanced in the tumor microenvironment by ATP/pH stimuli [267]. (**d**) PA signal could be significantly enhanced in tumor microenvironment by NO/pH stimulation [268]. (**e**) PET imaging enabled real-time monitoring of Enzyme/NIR/pH stimulus-responsive drug delivery systems [269]. (**f**) pH and NIR responsive system resulted in significant enhancement of CT contrast [270]. Reproduced with permission from the publishers of corresponding references.

Among the optical imaging methods, fluorescence imaging plays an important role because of its high resolution and low cost. For higher accuracy and specificity, optical imaging is also synergistically used with chemo-pharmacotherapy [262,263]. Huang et al. recently reported a pH responsive nanocarrier using NIR-II dye-based multifunctional telechelic glycopolymer (TTQ-TC-PFru). The NIR-II dyes in PFru-BTZ-PBOB nanoparticles could be used for optical imaging and photothermal therapy. Additionally, anticancer drug bortezomib (BTZ) was conjugated for chemotherapy. This novel NIR-II dye-based drug-loaded nanoparticles showed potential because of their precise targeting capability and the real time imaging modality to achieve better therapeutic effects [264]. In addition, a site-selective in situ growth-induced self-assembly method was reported recently. pH-responsive micelles were synthesized based on site-specific human serum albumin-poly(2-(diisopropylamino) ethyl methacrylate) (HSA-PDPA) conjugates. ICG could be loaded into the core of micelles. When present in the weak acid microenvironment in tumors, the nanoparticles rapidly decomposed into protonated moieties, allowing the fluorescence enhancement by 3–6 fold compared to non-stimulus-responsive nanoprobe and ICG [265] (Figure 14a).

US has become one of the most commonly used medical imaging techniques due to its excellent portability, noninvasiveness and low cost. However, ultrasound contrast imaging typically requires the use of diffusible microbubbles to diffuse into the surrounding media to enhance contrast. With the diffusion of gas, these microbubbles will be quickly cleared in vivo, for this reason the ultrasonic imaging has a shorter imaging time [266,271,272] (Figure 14b). In recent reports, a pH responsive solid ultrasound nanosensor (sUN) was developed that did not require the generation of bubbles. The sUN consists of three parts, a multi-empty silicone shell, a solid silica core and a pH-sensitive coating on its surface. This new type of sUN could expand and shrink responsive to the change of pH value owing to the pH-sensitive coating. When the concentration of hydrogen ions decreased, the ultrasound imaging was significantly enhanced [273].

Magnetic resonance imaging (MRI) is a powerful technology for diagnosis and bioimaging of diseases because of its non-invasiveness, reasonable imaging time, deep tissue penetration and high resolution [274,275]. Zhang et al. recently developed a triple stimulus (GSH/pH/NIR)-responsive nanocarrier based on magnetic hollow porous carbon nanoparticles (MHPCN). The outer shell of MHPCN consists of two layers: the inner layer with iron oxide (Fe_3_O_4_) as contrast agent for MRI and the outer layer with fluorescent carbon nanodots as the outer layer. DOX was selected as a model drug and folic acid (FA) was crosslinked for targeting purpose. This new triple stimulus-responsive nanodrug carrier can achieve synergistic photothermal/chemical therapy of tumor guided by MRI under the synergistic stimulation of low pH, high concentration of GSH and external NIR light in tumor cells [276]. In addition, a strategy using magnetic resonance/near-infrared fluorescence (MR/NIRFL) was developed to determine the location of tumors. Using pH and NIR as stimuli, chemo/photothermal therapy could be achieved for efficient cancer nanotheranostics [277]. Yang et al. used polyphenols as phase transfer agents to increase the solubility of hydrophobic magnetic nanoparticles. Meanwhile, polyphenols also promoted the self-assembly of nanoparticles. The core of this assembly is ATP/pH responsive for the dual imaging of MR/FL and photothermal therapy. When the nano-assembly entered the tumor microenvironment with lower pH value and higher concentration of ATP, they showed enhanced MR contrast (due to the strong binding affinity of ATP to Fe_3_O_4_) and significantly altered fluorescence signal (the protonation of hydroxyl groups in tannic acid (TA). Thus, functional activities in tumors could be tracked using this MR responsive nanoplatform [267] (Figure 14c).

Photoacoustic (PA) imaging is an emerging biomedical imaging modality with deep penetration depth, high spatial resolution, and high selectivity that combines the advantages of optical imaging and ultrasound imaging [278]. When laser irradiation is applied to biological tissues, PA signals are generated in the tissues, and the detailed information of human internal tissues could be collected and reconstructed in computer. Tan et al. designed a nitric oxide (NO)/pH activatable theranostic nanoprobe (DATN), taking advantage of high concentration of nitric oxide and low pH in tumor microenvironment. Benzo [C] [1,2,5] thiadiazole-5,6-diamine was used for the stimulus responsive π-receptor-π-donor (D-π-A-π-D) molecules. In the tumor microenvironment, DATN could turn on PA signals for tumor specific PA imaging in vivo. Using these dual stimuli, the PA signal intensity of DATN was 9.8 times higher than that NO responsive system and 132 times higher that of acid responsive system [268] (Figure 14d). Pu recently reported the synthesis of a fluoro-photoacoustic polymeric renal reporter (FPRR) for noninvasive near-infrared fluorescence (NIRF) and PA dual imaging of drug-induced acute kidney injury (AKI) in mice. FPRR consists of dextran (used to promote renal clarity), hemicyanine CyOH (used for imaging), and γ-glutamate (enzyme-responsive moiety). FPRR exhibited high renal clearance efficiency in living mice, it also enhanced NIRF and PA signals for real-time molecular imaging of AKI after the enzyme responsive moiety is cleaved. This nanoplatform showed potential for early detection of AKI in clinics [279].

Positron emission tomography (PET) can provide precise biological information on metabolic processes with whole-body penetration and excellent sensitivity at the molecular level using radiopharmaceuticals. It has been widely used in clinical disease treatment and diagnosis [280]. When used in combination with computed tomography (CT), the clinical effect of PET can be improved for disease diagnosis such as tumors and heart diseases [281]. For example, hyaluronic acid (HA)-functionalized MoS_2_ was designed with the surface modified by PEI and ^64^Cu (positron emitter with a half-life of 12.7 h). The resulting MoS_2_-PEI-HA multifunctional nanoplatform was endowed with the property of triple stimuli (pH/HAase/NIR) responsiveness. This new nanocarrier can be used as contrast agent for PET imaging, providing an effective way to monitor the treatment process and optimize disease management plan [269] (Figure 14e). Chen et al. developed a multimodal imaging synergistic therapy based on amphiphilic iron oxide-gold Janus nanoparticles (Fe_3_O_4_-Au-JNPs) in response to pH stimulus. It was shown that under the guidance of PET, MR and PA multimodal imaging, Fe_3_O_4_-Au-JNPs could effectively carry out ROS-mediated cancer treatment. After intravenous injection of [^64^Cu] radiolabeled JNP vesicles, PET imaging showed that the drug vesicles remained stable in the blood, but gradually dissociated in the liver, showing reduced hepatotoxicity. PET imaging modality provided effective real-time monitoring of the whole treatment process [282].

Computed tomography (CT) collects information of anatomical structures via X-ray scans and cross-sectional images are reconstructed by computer operation. Due to its penetration capability and fast scanning speed, CT is one of the most widely used clinical imaging methods. It is also usually used in combination with MRI, PA and other imaging modalities to achieve better imaging results. For example, as coronavirus disease 2019 (COVID-19) is spreading worldwide, CT has served as an important imaging tool for the assessment of COVID-19. CT scans can spot abnormalities of lungs such as multiple opacities (denser, more profuse and confluent) [283]. In other applications such as cancer diagnosis and treatment, CT also plays a significant role and is mostly used in combination with other imaging approaches. For example, Yu et al. recently developed bismuth nano-raspberries (Bi-BSA NRs) modified by BSA. Bi-BSA NRs have high drug loading efficiency (~69%) and can release the cargo in response to pH and NIR stimuli. The efficiency of CT contrast agent is improved by infrared thermal and PA. 100% tumor treatment rate was achieved by chemo-photothermal therapy. In conclusion, this Bi-BSA NRs have potential for the application of multimodality imaging and combination therapy [270] (Figure 14f).

## 4. Conclusions

In summary, nanotechnology-based therapeutics have developed rapidly in recent years, and a wide variety of novel strategies have been explored. Particularly, stimulus-responsive nano-systems warrant attention as they show promise in targeting and controlled release in response to endogenous or exogenous stimuli. Owing to their unique advantages, stimulus-responsive systems have been widely investigated as a strategy well-suited for nanotherapeutics. Furthermore, dual/multiple stimulus-responsive systems have been investigated for more accurate disease diagnosis and management. Self-immolative linkers with different cleavage and self-immolation rates hold potential as a versatile technology to address controlled drug release. Stimulus-responsive systems have shown promise for the treatment of various cancers, IBD, neuroinflammation, gastrointestinal diseases, bio-imaging and many others. However, the translational potential, especially for the highly complex chemically modified systems, is a concern. Drug-device combinations for external stimuli are more complicated than drug-alone approaches from regulatory perspectives. For example, for cancer treatment, stimulus-responsive systems treating only local tumors would require one to find advantages over the well-established modalities of surgery, radiotherapy and other ablation approaches, which do not have the complications of drugs. In addition, toxicity of not only the API, but also the carrier system, is another factor for consideration. Nevertheless, with smart and rational design, stimulus-responsive nanotechnology adds a new weapon to drug delivery and molecular imaging arsenal, and warrants further research and development.

## Figures and Tables

**Table 1:** Chemical structures for pH responsive systems.

Function Group	Mechanism	Ref
Imine	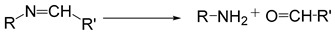	[34]
Hydrazone	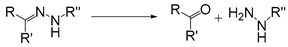	[35,36]
Ester	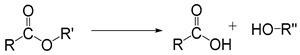	[37]
Acetal	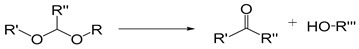	[38,39]
Cis-aconityl	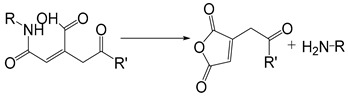	[40]
Orthoester	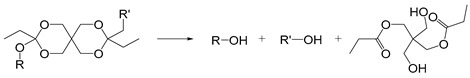	[41,42]
Silyl ether	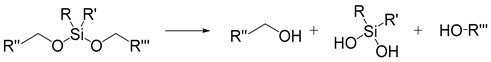	[43]

**Table 2:** Chemical structures for redox-responsive systems.

Function Group	Mechanism	Ref
Disulfide	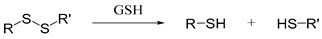	[53]
Diselenium	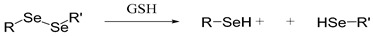	[54,55]
Thioether Selenium Tellurium	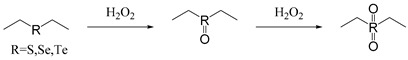	[56,57,58,59,60,61]
Oxalate ester	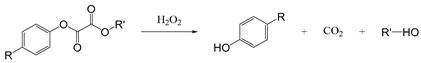	[62,63]
Vinyldithioether	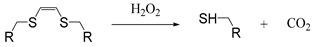	[64]
Thioketal	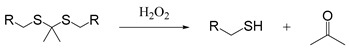	[65]
PBA/PBE	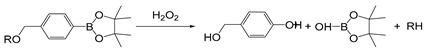	[66]

**Table 3:** Chemical structures for light-responsive systems.

Function Groups	Mechanisms	Ref
Anthracene	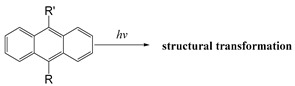	[110]
Coumarinyl ester	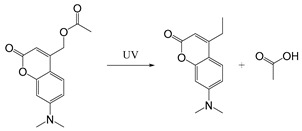	[111]
Arylmethyl	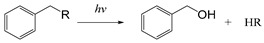	[112]
Pyrenylmethyl ester	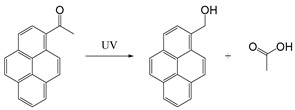	[113]
*O*-nitrobenzyl	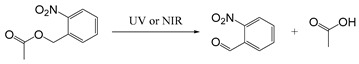	[114,115,116]
Azobenzene	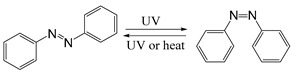	[120,121]
Spiropyan	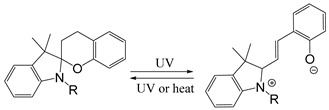	[122]
Diarylethene	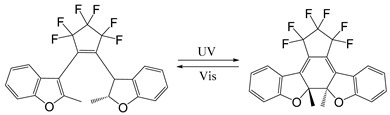	[123,124,125,126,127,128,129,130,131]

**Table 4:** Protection groups and stimuli of self-immolative linkers.

Structure Responsive to Stimulus	Stimulus	Triggers	Ref
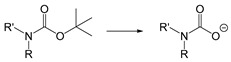	H^+^	pH	[176,177]
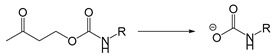	Piperidine/ Morpholine	pH	[178]
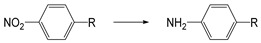	Zn/AcOH	Redox	[180]
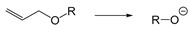	Pd (PPh_3_)_4_	Redox	[179,181]
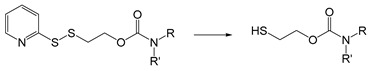	Dithiothreitol	Redox	[182,187]
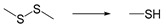	Thiols	Redox	[182]
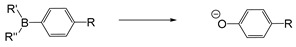	H_2_O_2_	Redox	[183,184,185,186]
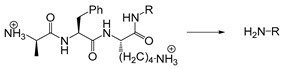	Plasmin	Enzyme	[172,188]
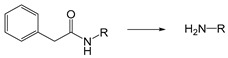	Penicillin-G-amidase (PGA)	Enzyme	[190]
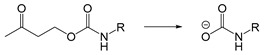	BSA	Enzyme	[189]
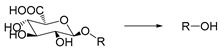	β-Glucuronidase	Enzyme	[191,192,193]
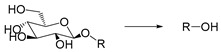	β-Galactosidase	Enzyme	[194]
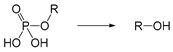	Alkaline phosphatase	Enzyme	[195,196]
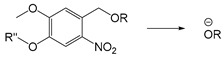	UV light/ NIR light	Light	[197,198,199,200]
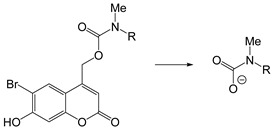	NIR light	Light	[201,202]

## Abbreviations

API	Active pharmaceutical ingredient
AAV2	Adeno-associated virus 2
APA	Apatite
ACP	Acetylated-chondroitin sulfate-protoporphyrin
BSA	Bovine serum albumin
CDDP	Cisplatin
CPT	Camptothecin
CS	Chitosan
CT	Computed tomography
COVID-19	Coronavirus disease 2019
DSS	Dextran sodium sulphate
DPPC	1,2-dipalmitoyl-sn-glycerin-3-phosphocholine
DOX	Doxorubicin
DAEs	Diarylethenes
Epo B	Epothilone B
FI	Fluorescence imaging
FA	Folic acid
FPRR	Fluoro-photoacoustic polymeric renal reporter
GSH	Glutathione
GIT	Gastrointestinal tract
GNS	Gold nanostars
GOx	Glucose oxidase
Hf	Hafnium
HPMCAS	Hydroxypropyl methylcellulose acetate succinate
HPPH	Hexyloxyethy-pyropheophorbide
ICG	Indocyanine green
IBD	Inflammatory bowel disease
Ins	Insulin
LCST	Lower critical solubility temperature
MRI	Magnetic resonance imaging
MTX	Methotrexate
MC	Merocyanine
MSNs	Mesoporous silica nanoparticles
MBs	Microbubbles
NCPs	Nanoscale coordination polymers
NQO1	NAD(P)H, quinone oxidoreductase isoenzyme I
Nap	Naproxen
NPCS	*N*-palmitoyl chitosan
NIPAAm	*N*-isopropylacrylamide
NIRF	Near-infrared fluorescence
NLC	Nanostructured lipid carrier
OEGMA	Poly (ethylene glycol) methyl ether methacrylate
OSPE	Organic semiconducting pro-nanoenzyme
OxiDEX	Oxidatively sensitive dextran
OPV	Oligo (p-phenylene vinylene) derivative
ONB	*O*-nitrobenzyl
OEG	Oligo (ethylene glycol)
PBA	Phenylboronic acid
PBE	Phenylboronic ester
PDT	Photodynamic therapy
PLGA	Poly (d,l-lactic-co-glycolic acid)
PEGDA	Poly (ethylene glycol) diacrylate
PS	Polystyrene
PEG	Polyethylene glycol
PEI	Polyethylenimine
PAMAM	Poly(amidoamine)
PNP-UC	PEG-NMAB-PLA-UCNPs
PTX	Paclitaxel
PDP	Pentadecylphenol
pTSL	Polymer-modified temperature-sensitive liposome
PAA	Propylacrylic acid
PAI	Photoacoustic imaging
PGA	Penicillin G amidase
PET	Positron emission tomography
P-gp	*P*-glycoprotein
ROS	Reactive oxygen species
RAFT	Reversible addition-break chain transfer
SPN	Semiconductor polymer nanoparticles
sPLA_2_	Phospholipase A2
sUN	Solid ultrasound nanosensor
SP	Spiropyran
TK	Thioketal
TL	Thioketal linker
TGMS	Triglyceride monostearate
TPA	Two-photon absorption
TSL	Temperature-sensitive liposomes
US	Ultrasound
UC	Upconversion
UCST	Upper critical solubility temperature
VEGF	Vascular endothelial growth factor
W/O/W	Water/oil/water
